# Partial proteomic analysis of brown widow spider (*Latrodectus geometricus*) venom to determine the biological activities

**DOI:** 10.1016/j.toxcx.2020.100062

**Published:** 2020-10-24

**Authors:** Pornsawan Khamtorn, Prapenpuksiri Rungsa, Nisachon Jangpromma, Sompong Klaynongsruang, Jureerut Daduang, Thanee Tessiri, Sakda Daduang

**Affiliations:** a Faculty of Pharmaceutical Sciences, Khon Kaen University, Khon Kaen, 40002, Thailand; b Protein and Proteomics Research Center for Commercial and Industrial Purposes (ProCCI), Khon Kaen University, Khon Kaen, 40002, Thailand; c Department of Clinical Chemistry, Faculty of Associated Medical Sciences, Khon Kaen University, Khon Kaen, 40002, Thailand; d Division of Pharmacognosy and Toxicology, Faculty of Pharmaceutical Sciences, Khon Kaen University, Khon Kaen, 40002, Thailand; e Center for Research and Development of Herbal Health Products (CDR-HHP), Faculty of Pharmaceutical Sciences, Khon Kaen University, Khon Kaen, 40002, Thailand

**Keywords:** Widow spider, *Latrodectus geometricus*, Venom, Toxicity, Hyaluronidase, Antibacterial activity, α-, β-, γ-, δ-, ε-LIT, alpha-, beta-, gramma-, delta-, epsilon-latroinsectotoxins, α-latrocrustotoxin, alpha-latrocrustotoxin, BWSV, brown widow spider venom, kDa, kilodalton, LC-MS/MS, liquid chromatography–mass spectrometry/mass spectrometry, OD_600_, optical density at 600 nm, PD_50_, 50% paralytic dose

## Abstract

Spiders use their venom for defence and to capture prey. These venoms contain a cocktail of biologically active compounds that display several different biological activities, such as large molecules and small molecules including peptides, proteins/enzymes, and other components. Thus, venom constituents have attracted the attention of biochemists and pharmacologists over the years. The brown widow spider (*Latrodectus geometricus*) is a venomous spider found worldwide, including in Thailand. This spider causes human injuries, and the venom has many potential applications. In this study, we investigated the complexity and pharmacology of brown widow spider venom. Spider crude venom was investigated using partial proteome techniques and enzymatic activity, toxicity, and antibacterial activity assessments. We found that crude venom displayed a wide range of molecular masses from 19 to over 97 kDa, with molecular masses of 66 kDa intensely stained. Peptides and proteins were identified by liquid chromatography-mass spectrometry/mass spectrometry (LC-MS/MS), which showed that the crude venom contained a variety of substances, including latrotoxins, apolipophorins, hemocyanins, chitinases, arginine kinase, allergen antigen 5-like protein, astacin-like metalloproteases, and serine proteases. High hyaluronidase activity was observed based on the turbidimetric method. The venom presented toxicity in crickets (PD_50_ = 0.73 ± 0.10 μg/g body weight), and substantial envenomation symptoms, such as slow-motion movement, paralysis, and even death, were noted. Moreover, this venom exhibited potential antibacterial activity against the gram-positive *Bacillus subtilis* but not the gram-negative *Pseudomonas aeruginosa*. Spider venom contains numerous molecules with biological activity, such as latrotoxins, which affect insects, and enzymes. In addition to latrotoxins, certain enzymes in venom are hypothesized to exhibit toxicity and antimicrobial activity. This study provides important information for the further development of natural compounds or insecticidal toxins.

## Highlights

• 8 proteins were identified using mass spectrometry.
• Venom exhibited potential hyaluronidase activity and antibacterial activity.
• Crude venom presented toxicity in crickets.
• The venom peptides can develop for a natural lead drug or an insecticidal toxin.

## 1 Introduction

Venomous animals use their venom for defence and prey capture. The venom contains a complex mixture both of organic and inorganic compounds, such as peptides and proteins, and mainly includes neurotoxins, enzymes, and other components that contribute to the venom functions (Saez et al., 2010). The composition of venom has attracted the interest of biochemists and pharmacologists over the years because the biologically active molecules in venom have potential applications. Although several bioactive components from venomous animals, such as snakes, scorpions, centipedes, cone snails, wasps, and spiders, have been studied, studies on widow spider venom remain limited.

The widow spider is a venomous animal that belongs to the phylum Arthropoda, class Arachnida, order Araneae, family Theridiidae, and genus *Latrodectus* (Yan and Wang, 2015). This genus contains 32 species (World Spider Catalog, 2020), including black widow spiders, which are known for their venom that contains neurotoxins. *Latrodectus* spp. are called widow spiders because the female spider eats the male spider after mating. The brown widow spider, *Latrodectus geometricus* has an orange hourglass on its ventral surface, whereas other species have a red hourglass. Geographically speaking, brown widows are found across the globe (exception of the polar regions) (Choi et al., 2019; Muslimin et al., 2015) and commonly inhabit garages, agricultural areas, and even houses. Unsurprisingly, numerous reports have described the frequency of spider bites and envenomation worldwide (Jelinek, 1997). Spider bites cause local and systemic symptoms, including paralysis, immobilization, sharp pain, diaphoresis, hypertension, headache, and numbness (Duan et al., 2008).

The components of brown widow venom cause massive transmitter release from presynaptic neuron cells by binding receptors, which stimulates both the presence and absence of calcium ions (Ca^2+^) in neuron cells. Latrotoxins are major toxins present in venom. Seven different latrotoxins have been isolated from black widow spiders (*Latrodectus mactans*) (Krasnoperov et al., 1990), and they have specific targets. Specifically, α-latrotoxin targets vertebrates, latroinsectotoxins (LITs; α-, β-, γ-, δ-, ε-LIT) target insects, and α-latrocrustotoxin targets crustaceans. α-Latrotoxin acts in vertebrates via both the presence and absence of calcium ions, causing massive neurotransmitter release in neuronal cells. In addition, low molecular weight proteins (LMWPs and LMWP2) have been isolated from the venom and are called latrodectins. These proteins contribute to the neurotoxicity of latrotoxins by increasing their binding to membrane targets (Grishin et al., 1993). In the 2000s, several reports highlighted severe cases of envenomation. In one case report, patients that were bitten by the brown widow spider initially presented local symptoms, such as erythema, oedema, swelling, numbness, and pain at the bite site. After 20 min, the erythema and oedema expanded and the patient started to experience headache, cramps, vomiting, debilitating pain, and fasciculations within 30 min. These symptoms were alleviated after 6 h, and the patient recovered over the following 24 h. In addition to previously reported local symptoms, local burning was also reported during brown widow spider envenomation (Almeida et al., 2009; Goddard et al., 2008; Shackelford et al., 2015).

In the present study, we performed biochemical analyses and antibacterial assays of venom from the brown widow spider *L. geometricus*. The results indicated that this spider venom contains several compounds and exhibits antibacterial activity, and research on its venom has potential application for the development of peptides as pharmaceuticals and bioinsecticide agents.

## 2 Materials and methods

### 2.1 Chemicals

Ammonium bicarbonate (AmB); Bradford reagent; hyaluronidase from bovine testes; *N,N,N’N’*-tetramethylenediamine (TEMED); iodoacetamide (IAA); and trifluoroacetic acid (TFA) were obtained from Sigma-Aldrich, USA. Sodium dodecyl sulphate (SDS) was obtained from Lobal Chemie, India. Acetonitrile (ACN) was obtained from VWR Chemicals, France. An Amersham™ LMW Calibration Kit for SDS Electrophoresis as a protein standard was obtained from GE Healthcare, UK. Cetyltrimethylammonium bromide (CTAB) was obtained from Panreac AppliChem, Germany. Dithiothreitol (DTT) was purchased from Vivantis, Malaysia. Formic acid was obtained from Fluka, Switzerland.

### 2.2 Spider venom collection

Brown widow spiders (*L. geometricus*) were obtained from suburban areas of Khon Kaen city, Khon Kaen Province. All animals received food and were housed under controlled environmental conditions. Briefly, spiders were housed in plastic boxes and fed crickets (*Gryllus* sp.) twice per week for 3 months under the appropriate conditions (27 ± 1 °C, 60–70% R.H., photoperiod of light:dark cycle 16:8 h) to ensure that adult spiders were used in the studies. Adult female brown widow spiders were not allowed to feed for 1 week before venom collection. For venom collection, the spider venom was obtained using the cold shock method at 4 °C for 1 h followed by −20 °C for 30 min. The venom droplets were collected in a capillary tube that was placed in a microcentrifuge tube that was kept on ice (Khan et al., 2013). Venom was lyophilized and stored at −80 °C until use. The protein content in venom was measured using the Bradford method, and bovine serum albumin (BSA) was used as a standard. An EnSight Multimode Plate Reader (PerkinElmer, USA) was used for the assay, and concentration was expressed as microgram per microliter (μg/μl).

### 2.3 Sodium dodecyl sulphate-polyacrylamide gel electrophoresis (SDS-PAGE) and de-staining solution

SDS-PAGE of crude venom was performed under reducing conditions according to the method of Laemmli (1970). The lyophilized venom was dissolved in Milli-Q water and analysed using 13% SDS-PAGE. An AmershamTM LMW Calibration Kit for SDS Electrophoresis (GE Healthcare, UK) was used as the standard protein, and the SDS-PAGE gel was visualized using Coomassie brilliant blue G-250 staining.

### 2.4 Purification of spider venom using reverse-phase high-performance liquid chromatography

Lyophilized crude venom (2 mg) was dissolved in 200 μl of 0.1% trifluoroacetic acid (TFA) in water. Debris was removed by centrifugation at 10,000×*g* for 2 min. Separation was performed using a RP-HPLC C18 (0.46 × 25 cm, 5 μm particle size) column. Proteins and/or peptides were eluted at 1 ml/min with a linear gradient of 0.1% TFA in water (solvent A) and 0.085% TFA in acetonitrile (ACN) (solvent B) under the following conditions: 0% B for 10 min, 5–30% over 10 min, 30–50% over 10 min, 50–70% over 40 min, and 70–100% over 10 min. The absorption of proteins and/or peptides was monitored at 214 nm. Then, the fractions were manually collected, lyophilized, and resuspended in sample buffer. SDS-PAGE was performed under reducing conditions, and protein bands were stained with Coomassie brilliant blue G-250 staining before mass spectrometry analysis. Since the RP-HPLC fraction had a low protein concentration, silver staining was used for visualization.

### 2.5 In-gel digestion with trypsinization

Protein bands were excised from the SDS-PAGE gel and washed three times (10 min, with shaking each time) with 200 μl of deionized water and then AmB, AmB/ACN (1:1) and ACN, and gel bands were dried for 5 min. Subsequently, 100 μl of 10 mM DTT/20 mM AmB was added. Following incubation at 56 °C for 45 min, 100 μl of 55 mM iodoacetamide (IAA)/20 mM AmB was added, and incubation was continued in dark conditions at room temperature for 30 min. A 200 μl aliquot of 20 mM AmB/ACN (1:1) was then added, and the tube was shaken for 10 min (repeated once). Then, 200 μl of ACN was added and shaken for 10 min. Gel bands were dried for 5 min, and then 20–40 μl of freshly prepared enzyme solution (trypsin) was added, and the tube was incubated at 4 °C for 30 min. After liquid phase removal, 5–10 μl of 25 mM AmB was added and incubated at 37 °C overnight. Then, 50 μl of 20 mM AmB, extraction buffer 1 and extraction buffer 2 were added sequentially, the tube was shaken for 10 min, and the liquid phase was retained following each addition of solution. The mixture was dried in a SpeedVac Vacuum Concentrators (Thermo Scientific, USA) at 45 °C for 120 min. The pellet was collected, and then 30–40 μl of resuspension buffer (ACN and formic acid) was added to the pellet. The solution was centrifuged at 10,000 rpm at room temperature for 10 min before being injected into an LC-MS instrument or kept at −70 °C until used (Heawchaiyaphum et al., 2018).

### 2.6 Protein identification with LC-MS/MS and data search

For the LC-MS/MS analysis, the samples were analysed using a nano-liquid chromatography system (EASY-nLC II, Bruker) coupled to a Q-TOF mass spectrometer (MicrOTOFQ-II, Bruker) equipped with an ESI nano-sprayer. The instrument was maintained and operated at the Khon Kaen University Research Instrument Center, Thailand. The ESI-Q-TOF instrument was calibrated in the m/z range of 50–3000 using an internal calibration standard (Tune mix solution), which was supplied by Agilent. Sample volumes of 3–6 μl were loaded by the autosampler onto an EASY-Column (10 cm, ID 75 μm, 3 μm, C18-A2; Thermo Scientific, USA) using a flow rate of 300 nl/min and a linear gradient from Solution A (0.1% formic acid) to 45% Solution B (0.1% formic acid in ACN) over 45 min. The Bruker Daltonics software package HyStar v.3.2 was used to control the Q-TOF device. LC–MS/MS spectra were analysed using Compass Data Analysis v.4.0.

Protein identification was performed by searching against the NCBIprot (Other Metazoa) protein database using the MASCOT MS/MS Ion Search program (www.matrixscience.com) with the initial searching parameters: enzyme: trypsin; fixed modification: carbamidomethylation (C); variable modification: oxidation (HW) and oxidation (M); peptide mass tolerance: 0.5 Da; fragment mass tolerance: 0.5 Da; peptide charge states: +1, +2, and +3; instrument type: ESI-TRAP; and report top: auto.

### 2.7 Toxicity

Adult crickets (*Gryllus* sp.) (average bodyweight, 0.27 g) were intraperitoneally injected with 20 μl of phosphate-buffered saline (PBS) with or without venom. After injection, these crickets were placed in an overturned position in the plastic container, and the mortality, paralysis, and behaviours were monitored within 30 min post-injection. The assay endpoint was the calculation of the 50% paralytic dose (PD_50_). The PD_50_ was evaluated using a range of 6 venom doses (0, 0.2, 0.5, 0.8, 1, and 2 μg/g body weight), with 6 crickets per dose (n = 6). Venom-injected crickets that could not turn to a righted position were paralyzed (Uawonggul et al., 2014).

### 2.8 Hyaluronidase activity

Hyaluronidase activity was measured using the turbidimetric method reported by Villas-Boas et al. (2012), with slight modifications. Briefly, various concentrations of brown widow spider venom (BWSV) (0.21, 0.42, and 0.83 μg/μl) in a volume of 5 μl were mixed with 25 μl of 0.5 mg/ml hyaluronic acid (HA) and acetate buffer (0.2 M sodium acetate, pH 4.0, with 0.15 M NaCl) to a final volume of 50 μl, and this reaction mixture was placed in a microplate. The reactions mixtures were incubated at 37 °C for 30 min. Then, 100 μl of 2.5% CTAB in 2% NaOH was added to stop the reaction. Milli-Q water was used as a blank, and 0.67 μg/μl of hyaluronidase from bovine testes was used as a positive control. The solutions were read at 405 nm in the microplate reader. The specificity was expressed as turbidity reduction units per microgram (TRU/μg).

### 2.9 Antibacterial activity

Gram-positive (*Bacillus subtilis*) and gram-negative (*Pseudomonas aeruginosa*) bacteria were used to investigate the antibacterial activity using the disc diffusion assay. Bacteria were cultured in nutrient broth (NB) medium to the exponential phase (OD_600_ = 0.5) and swabbed on nutrient agar (NA). Fifteen microliters of venom solution (0, 10, and 30 mg/ml) was dropped onto Whatman filter paper discs (6 mm in diameter) and subsequently placed on NA. The plates were incubated at 37 °C for 24 h to observe the inhibition zone. Ampicillin and streptomycin (10 μg/paper disc (Oxoid, England)) were used as a positive control, and Milli-Q water served as a negative control. Antibacterial activity was evaluated by measuring the inhibition zone diameter in millimetres using a scale. All experiments were performed in triplicate (Lei et al., 2015).

### 2.10 Statistical analysis

The results were expressed as the mean ± standard error of the mean (S.E.M.). All data were analysed by one-way analysis of variance (ANOVA) followed by Tukey's post hoc test using Statistical Package for Social Studies (SPSS) IBM version 22.0 (Armonk, USA). *P* < 0.05 was considered statistically significant.

## 3 Results and discussion

### 3.1 SDS-PAGE of spider venom and LC-MS/MS identification of its separated proteins

The lack of protein information from spider venom has limited our detailed knowledge of brown widow spiders. Although the protein composition of *Latrodectus tredecimguttatus* venom has been clarified to some extent by a partial proteomic investigation (Duan et al., 2008), the venom from *L. geometricus* has remained poorly understood. Therefore, in this work, we investigated adult female *L. geometricus* venom, which was collected using the cold shock method, using proteomic techniques. The collected venom was not contaminated with other components because the spiders were not allowed to feed for 1 week prior to venom collection. Thus, the venom was pure and appropriate for protein identification.

BWSV was investigated by 13% SDS-PAGE performed at 150 V for 120 min to assess the pattern of protein separation. Venom proteins exhibited a wide range of molecular masses from approximately 19.0 to 97.0 kDa and greater than 97.0 kDa. Major protein bands were present at 66.0, 70.0 and over 97 kDa, which were similar to results noted for *L. tredecimguttatus* venom (43–120 kDa) (Yan et al., 2014). Thus, the venom proteins include large proteins and low-molecular weight proteins or peptides.

**Fig. 1:**
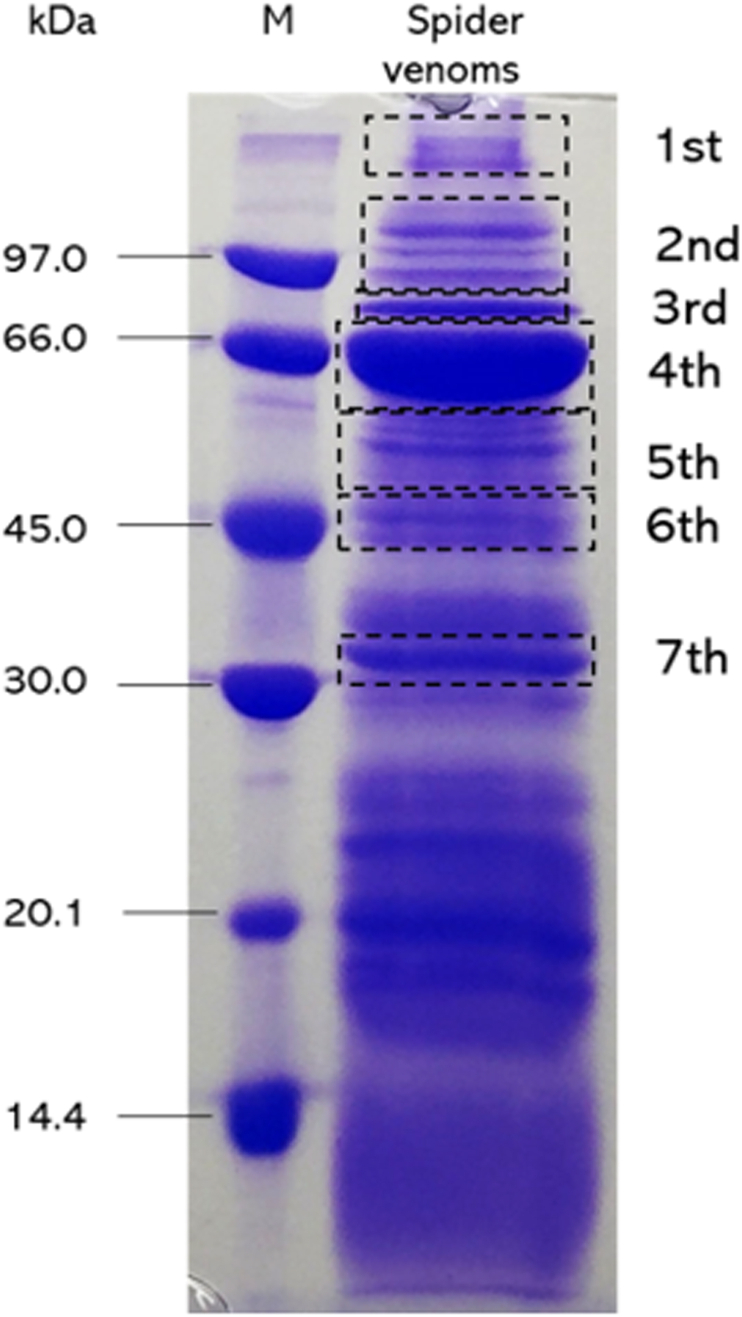
SDS-PAGE analysis of crude brown widow spider venom performed under reduced conditions. Seven bands of spider venom were excised for protein identification by LC-MS/MS (M = protein marker; sample venom = protein of BWSV, 10 μg of protein).

To identify the proteins, protein bands were divided into the 1st-7th bands according to obvious separation, mostly high molecular weight proteins were identified, and low molecular weight proteins were not chosen because of overlapping proteins (Fig. 1). These bands were analysed and identified using LC-MS/MS. The results showed that the first band corresponded to apolipophorins and that the second band presented 5.2% sequence coverage with latrotoxins, including latroinsectotoxin, latrocrustatoxin, and latrotoxin. In addition, the electrophoretic pattern of the venom also showed proteins ranging from 110 to 130 kDa, which correspond to the molecular weight of latrotoxins, a major toxin group in the genus *Latrodectus* (Grishin et al., 1993; Yan and Wang, 2015). According to de Roodt et al. (2017), latrotoxins present an important percentage of the total venom weight, suggesting that *L. geometricus* venom is rich in toxins. Interestingly, the third and fourth bands corresponded to hemocyanins, which are components of secretory epithelial cells of the venom glands and found in the blood and lymphatic fluids of many arthropod species, including spiders (Smith and Russell, 1966). These results suggested that the proteins were not contaminants from body fluids. In addition, the fifth and sixth bands were correlated with enzymes, i.e., chitinase, astacin-like metalloprotease and arginine kinase. The fifth band presented one unique peptide matched with the chitinase of *Araneus ventricosus*. The sixth band showed 40% sequence coverage of astacin-like metalloprotease toxin, and 5 unique peptides matched with arginine kinase. These enzymes are also found in other venoms, such as that of *Podocoryne carnea* (Pan et al., 1998) and *Loxosceles intermedia* (da Silveira et al., 2006). Finally, the seventh band presented partial coverage of serine protease peptide, and 3 unique peptides were matched (Table 1). Duan et al. (2006) reported that some proteases, such as astacin-like metalloprotease, serine protease, and antigen 5-like protein, are related to the noxious effects of *L. tredecimguttatus* and *Loxosceles* venoms. All peptide sequences are available in Supplementary Table 1.

**Table 1:** Protein identification of *Latrodectus geometricus* venom using SDS-PAGE.

Bands	Protein name	Accession no.	Theoretical MW (kDa)^a^	Number of unique peptides matched	Protein sequence coverage (%)	Score XC^b^
1st	Uncharacterized protein LOC107439767 of *Parasteatoda tepidariorum*	XP_015907950.1	212.279	6	4.5	263
1st	Apolipophorins of *Parasteatoda tepidariorum*	XP_021003806.1	366.911	2	0.6	94
2nd	α-latroinsectotoxin precursor of *Latrodectus tredecimguttatus*	CAA78464.1	158.449	8	5.2	287
2nd	δ-latroinsectotoxin precursor of *Latrodectus tredecimguttatus*	CAA63363.1	136.137	4	3.9	134
2nd	α-latrocrustotoxin precursor	Q9XZC0.2	158.752	3	2.9	111
2nd	α-latrotoxin of *Latrodectus hesperus*	AGD80166.1	154.923	4	3.8	97
2nd	α-latrotoxin precursor of *Latrodectus tredecimguttatus*	CAA38753.1	157.386	4	3.6	73
3rd	Hemocyanin C chain of *Latrodectus hesperus*	ADV40153.1	37.976	12	52.0	410
3rd	Hemocyanin C chain of *Parasteatoda tepidariorum*	XP_015914467.1	78.587	13	18.7	377
3rd	Hemocyanin C chain of *Stegodyphus mimosarum*	KFM75964.1	72.640	7	9.4	204
3rd	Hemocyanin F chain of *Stegodyphus mimosarum*	KFM66956.1	72.541	7	11.1	158
3rd	Hemocyanin subunit F of *Nephila inaurata madagascariensis*	CAD68056.1	72.326	6	9.9	152
3rd	Hemocyanin subunit D of *Nephila inaurata madagascariensis*	CAD68054.1	72.483	4	6.4	117
3rd	α-latroinsectotoxin precursor of *Latrodectus tredecimguttatus*	CAA78464.1	158.449	4	2.9	113
3rd	Hemocyanin subunit F of *Euphrynichus bacillifer*	CCA94920.1	72.485	4	4.8	108
3rd	Hemocyanin subunit D of *Latrodectus hesperus*	ADV40138.1	38.235	3	10.5	103
3rd	Hemocyanin G chain of *Stegodyphus mimosarum*	KFM73503.1	69.722	3	4.9	88
3rd	Hemocyanin AA6 chain	P80476.1	72.197	4	6.2	67
3rd	Hemocyanin B chain of *Stegodyphus mimosarum*	KFM59357.1	74.335	3	4.0	57
4th	Hemocyanin F chain of *Parasteatoda tepidariorum*	XP_015923305.1	66.818	3	5.3	226
5th	Chitinase of *Araneus ventricosus*	AAN39100.1	47.608	1	2.3	60
6th	Astacin-like metalloprotease toxin of *Stegodyphus mimosarum*	KFM58572.1	46.075	11	40	361
6th	Astacin-like metalloprotease toxin 1 of *Parasteatoda tepidariorum*	XP_015917054.1	46.392	9	29.5	281
6th	Astacin-like metalloprotease toxin 1 of *Parasteatoda tepidariorum*	XP_021000929.1	45.765	7	22.6	232
6th	Arginine kinase of *Parasteatoda tepidariorum*	XP_015928377.1	46.76	5	14.2	138
7th	Hemocyanin subunit D of *Latrodectus hesperus*	ADV40138.1	38.235	5	17.4	225
7th	Hemocyanin subunit C of *Latrodectus hesperus*	ADV40153.1	37.976	2	8.2	70
7th	Putative serine protease of *Latrodectus hesperus*	ADV40282.1	38.728	3	7.4	57

a Theoretical molecular weight (MW) obtained after LC-MS/MS analysis.

b Score XC obtained after LC-MS/MS analysis.

### 3.2 RP-HPLC and LC-MS/MS analysis

**Fig. 2:**
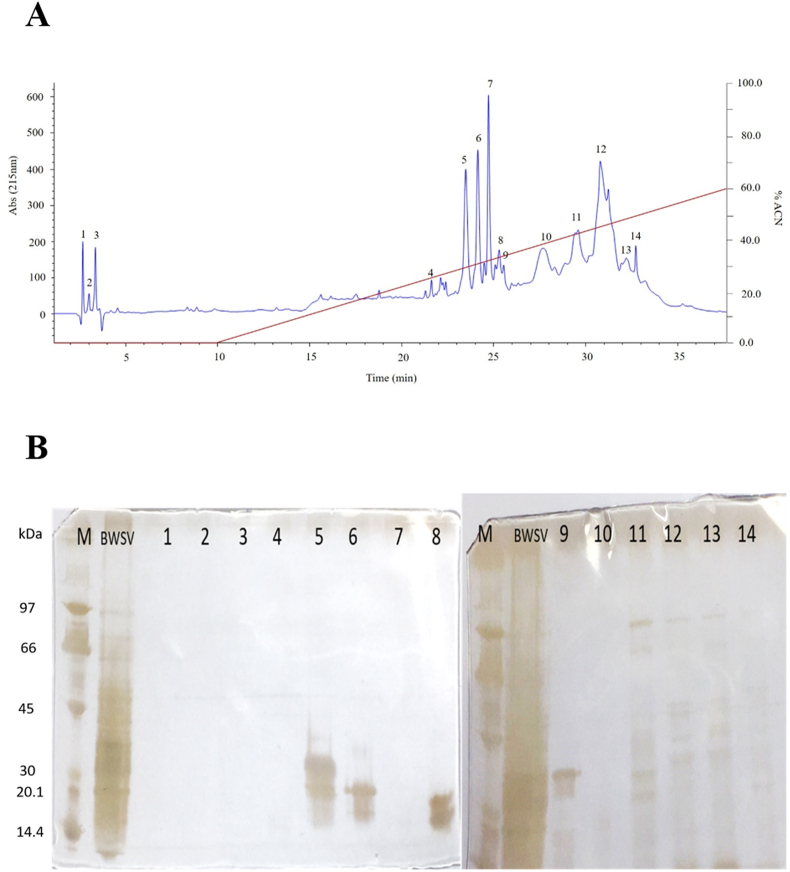
RP-HPLC of BWSV. Here, 2 mg of purified protein from BWSV was fractionated by RP-HPLC. The fractions were manually collected, and SDS-PAGE was performed. Protein bands containing low protein concentrations were visualized by silver staining. For analysis by LC-MS/MS, gel bands were stained with Coomassie brilliant blue. (A) RP-HPLC of BWSV was performed on a C18 column, and proteins were eluted with an acetonitrile (ACN) gradient at 1 ml/min. Fourteen fractions were eluted from crude BWSV. (B) SDS-PAGE of BWSV fractions after RP-HPLC separation.

BWSV was investigated using RP-HPLC with a linear gradient of 0.1% TFA in water (solvent A) and 0.085% TFA in acetonitrile (ACN) (solvent B), and the fractions were manually collected. Fig. 2 presents the 14 collected fractions. The gel showed a wide range of molecular weights (15–97 kDa). The following peaks were noted in the fractions: peak number 5 was 20–35 kDa, peak number 6 was 15–25 kDa, peak number 8 was 15–23 kDa, peak number 9 was 20–35 kDa, and peak numbers 11–14 were 20–97 kDa. Protein bands were excised from the gel and analysed with LC-MS/MS (Table 2). The protein and peptides corresponded to those identified above. However, we identified an additional protein, antigen 5-like protein in the crude spider venom. All protein sequences are available in Supplementary Table 2.

**Table 2:** Protein identification of *Latrodectus geometricus* venom using RP-HPLC fraction.

Fraction no.	Protein name	Accession no.	Theoretical MW (kDa)^a^	Number of unique peptides matched	Protein sequence coverage (%)	Score XC^b^
5	Astacin-like metalloprotease toxin 1 of *Parasteatoda tepidariorum*	XP_015917054.1	46.392	9	29.5	281
6	Arginine kinase of *Parasteatoda tepidariorum*	XP_015928377.1	46.76	4	10.33	138
8	Putative serine protease of *Latrodectus hesperus*	ADV40282.1	38.728	3	7.4	57
8	Chitinase of *Araneus ventricosus*	AAN39100.1	47.608	1	2.3	60
9	Astacin-like metalloprotease toxin of *Stegodyphus mimosarum*	KFM58572.1	46.075	11	40	361
11	α-Latroinsectotoxin precursor of *Latrodectus tredecimguttatus*	CAA78464.1	158.449	8	5.2	287
11	U24-ctenitoxin-Pn1a of *Trichonephila clavipes*	PRD29014.1	116.247	6	40.6	100
12	Venom allergen antigen 5-like protein of *Dirofilaria immitis*	AAB62535.1	56.6291	1	4.1	54
12	δ-Latroinsectotoxin precursor of *Latrodectus tredecimguttatus*	CAA63363.1	136.137	4	3.9	134
13	α -Latrocrustotoxin precursor of *Latrodectus tredecimguttatus*	Q9XZC0.2	158.752	3	2.9	111
13	α -Latrotoxin of *Latrodectus hesperus*	AGD80166.1	154.923	4	3.8	97
14	α -Latrotoxin precursor of *Latrodectus tredecimguttatus*	CAA38753.1	157.386	4	3.6	73

a Theoretical molecular weight (MW) obtained after LC-MS/MS analysis.

b Score XC obtained after LC-MS/MS analysis.

### 3.3 Effect of venom on crickets

Venom dissolved in PBS was intraperitoneally injected into crickets. A few minutes after venom injection, crickets presented with neurological disturbances in motion behaviours, such as slow-motion movement, constant twitching in the terminal portions of all legs, paralysis, and inability to overturn to a normal position for 30 min to 1 h. The crickets subsequently died after 24 h. These signs were obvious and consistent with neurotoxic symptoms in crickets. However, crickets in the control group did not show any neurotoxic symptoms after intraperitoneal injection. The PD_50_ value was 0.73 ± 0.10 μg/g body weight. Spider venom is released to prey and protects spiders, and many reports suggest that venom acts as a neurotoxin in animals and humans. According to L. *geometricus* crude venom shown the PD_50_ 30 min after injection was 0.73 ± 0.10 μg/g body weight. In addition, Zobel-Thropp et al. (2014) summarized the paralytic dose (PD_50_) values from bioassays using spider venoms in various target species and different injection methods. However, our data can be compared only with the results from other spider venoms when the relative units are the same (μg/g). The potency of *L. geometricus* (0.73 μg/g) venom is greater than that of *Loxosceles arizonica* (2.6 μg/g), *Sicarius* cf. *hahni* (1.5 μg/g), *S. rugosa* (3.2 μg/g) (Zobel-Thropp et al., 2010), *Plectreurys tristis* (3.3 μg/g) (Zobel-Thropp et al., 2014), *Phoneutria nigriventer* (43.5 μg/g), and *Nephilengys cruentata* (24.6 μg/g) (Manzoli-Palma et al., 2003). Regarding venom potency, crickets exhibited paralytic signs within 30 min after injection of low doses of the venom and presented paralysis and even death 1 h after injection with higher doses of the venom. Information on venom potency is important when testing individual neurotoxins from *L. geometricus* on specific neurophysiological targets. The MS analysis revealed the presence of the latrotoxin family, especially insectotoxins. Thus, it is possible that these proteins participate in the paralysis observed in crickets.

### 3.4 Hyaluronidase activity

**Fig. 3:**
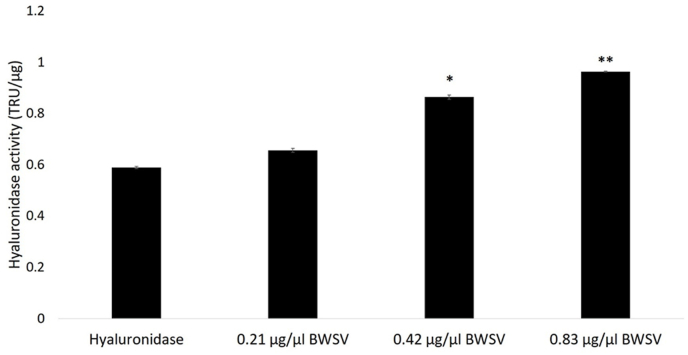
Hyaluronidase activity of *Latrodectus geometricus* venom (BWSV). The hyaluronidase activity is expressed in turbidity reduction units (TRU). The results are presented for triplicate experiments and expressed as the specific activity (TRU/μg) ± S.E.M. Bovine testicular hyaluronidase (0.67 μg/μl) was used as the positive control. *Indicates a statistically significant difference (*p* < 0.05) compared to control group (hyaluronidase).

The hyaluronidase activity of spider venom was assessed using the turbidity assay. Spider venom retained activity after incubation with hyaluronidase. The solution cleared in a dose-dependent manner (data not shown). The hyaluronidase activity results are presented in Fig. 3. Hyaluronidases are a group of enzymes found throughout the animal kingdom, including venomous animals. This enzyme hydrolyses hyaluronic acid, which is a component of the extracellular matrix; thus, hyaluronidase refers to a spreading factor (Girish and Kemparaju, 2007). Hyaluronidase activity has been found in the venom of numerous spiders, such as *Phoneutria nigriventer*, *Loxosceles reclusa* (Wright et al., 1973), *Hippasa partita*, *H. lycosina* and *H. agelenoides* (Nagaraju et al., 2007). Moreover, Ferrer et al. (2013) reported that recombinant hyaluronidase increases the dermonecrotic effect.

The spreading property of the enzyme is hypothesized to facilitate the spreading of the toxin from the bite site to the circulatory system. In this experiment, hyaluronidase activity was assessed by measuring the reaction turbidity using various concentrations of spider venom and hyaluronic acid as a substrate. The result showed that hyaluronidase activity increased in a dose-dependent manner (Fig. 3) (*p* < 0.05). Spider venom is composed of many biological molecules, such as peptide toxins from the latrotoxin family, which are major neurotoxins of the brown widow spider. Individuals with spider bites exhibit local symptoms, such as erythema, swelling, pain at the bite site, and headache. We did not find hyaluronidases in the proteomic analysis because they are not a major component of venom. Nevertheless, we found hyaluronidase sequences in the transcriptomic analyses of other studies (data not shown). Hyaluronidases are a spreading factor that help toxins spread in victims, thus leading to the appearance of these symptoms.

### 3.5 Antibacterial activity

In the present study, the antibacterial activity of the crude spider venom against gram-positive and gram-negative bacterial strains was assessed using agar-disc diffusion assays. The inhibition zone diameter (IZD) values are shown in Table 3. As shown in the table, 30 μg of crude spider venom exhibited inhibitory activity against gram-positive bacteria (*B. subtilis*), with an IZD of 9.33 ± 0.5 mm, whereas another concentration of crude spider venom did not inhibit bacteria. No inhibition zone was noted for gram-negative (*P. aeruginosa*) bacteria (data not shown). Ampicillin and streptomycin (10 μg/disc), which were used as positive controls, exhibited a clear zone of inhibition on the plates. Venoms from other spider species also inhibited the bacterial stains. For example, hemolymph of *Agelena labyrinthica* inhibited both of gram-positive and gram-negative bacteria, including *B. subtilis*, *P. aeruginosa*, *Shigella* sp., *Escherichia coli*, and *Staphylococcus aureus*, as assessed by agar-disc diffusion (Yiğit and Benli, 2008), and purified spider venom (*Crossopriza lyoni*) showed broad-spectrum activity and exhibited large inhibition zones via agar disc diffusion (Gupta and Upadhyay, 2016). BWSV did not inhibit gram-negative bacteria because the crude venom contains complex components that may interfere with bacterial membrane disruption.

**Table 3:** Antibacterial activity of crude spider venom.

Against to *Bacillus subtilis*	Inhibition zone diameter (mm)
1) Brown widow spider venom 30 μg	9.33 ± 0.5
2) Brown widow spider venom 20 μg	N/A
3) Brown widow spider venom 10 μg	N/A
4) Milli-Q water	N/A
5) Ampicillin	29 ± 1
6) Streptomycin	20.67 ± 0.6

Spider venom consists of an abundance of small polypeptides, such as antimicrobial peptides (AMPs) (Vassilevski et al., 2009). AMPs disrupt structural and functional cellular components that are essential for pathogen survival by directly binding the cell membrane, even at micromolar concentrations (Jenssen et al., 2006). Previous reports have described antimicrobial peptides in spider venom. For example, lycotoxin I and II were isolated from *Lycosa carolinensis* and exhibit amphipathic α-helix structures for pore-forming activity (Yan and Adams, 1998), and Lycosin-I is a cationic peptide isolated from *Lycosa singorensis* that showed broad-spectrum antibacterial activity (Tan et al., 2013). Spider venom contains AMPs, and these molecules play a role in spider-prey interactions, including prey capture and protection from ingesting infectious organisms. In another study, we identified the activities of peptides of *L. geometricus* venom, such as anti-Alzheimer's disease peptides, anticancer peptides, and some antimicrobial peptides (data not shown); however, this venom did not present activity against gram-negative bacteria in this study. Therefore, we reviewed the literature and got the result from our transcriptomic study to predict that BWSV has antibacterial activity. The venom exhibited neurotoxic effects against insects, as well. The potency will be more informative when testing individual neurotoxins from *L. geometricus* on specific neurophysiological targets. Moreover, further experiments could identify peptides for drug development.

## 4 Conclusions

BWSV contains many biologically active compounds. The biological molecules in venom have been hypothesized to be neurotoxins, and some enzymes have important roles in venom toxicity. The effects of individual neurotoxins from *L. geometricus* on specific neurophysiological targets should be assessed in further studies. Specifically, antimicrobial peptides and potential insecticides in spider venom should also be assessed in further experiments, as well. In addition, the enrichment of active compounds has the potential for further development and could lead to the discovery of new compounds or insecticides.

## Ethical statements

A manuscript entitled **“Partial proteomic analysis of brown widow spider (*Latrodectus geometricus*) venom to determine the biological activities**” by Pornsawan Khamtorn, Prapenpuksiri Rungsa, Nisachon Jangpromma, Sompong Klaynongsruang, Jureerut Daduang, Thanee Tessiri, Sakda Daduang has been performed according to the international, national and institutional rules considering animal experiments, clinical studies and biodiversity rights. I declare the originality of all experiments in this manuscript with full acknowledgement of the works and ideas of any other people who are cited in the article without conflicts of interest. This manuscript has not been submitted elsewhere for publication.

## CRediT author statement

**Pornsawan Khamtorn:** conducted all the experiments, Formal analysis, Writing - original draft, Writing - review & editing. **Prapenpuksiri Rungsa:** Methodology. **Nisachon Jangpromma:** partially contributed bacteria, amino acid sequencing, bioinformatics. **Sompong Klaynongsruang:** partially contributed bacteria, amino acid sequencing, bioinformatics. **Jureerut Daduang:** partially contributed bacteria, amino acid sequencing, bioinformatics. **Thanee Tessiri:** supported the HPLC training, Writing - review & editing, partially help to corresponded with the journal editor. **Sakda Daduang:** Conceptualization, Writing - review & editing, corresponded with the journal editor.

## Declaration of competing interest

The authors declare that they have no known competing financial interests or personal relationships that could have appeared to influence the work reported in this paper.
