# Supplementary material for: Partial proteomic analysis of brown widow spider (Latrodectus geometricus) venom to determine the biological activities
Source: Toxicon X. 2020 Oct 24;8:100062. doi: 10.1016/j.toxcx.2020.100062 (PMC7607507; doi:10.1016/j.toxcx.2020.100062)
Supplement: Multimedia component 1 [file mmc1.docx]

Supplementary Table 1. Protein identification of *Latrodectus geometricus* venom using SDS-PAGE

| Bands | Protein name | Accession no. | Theoretical MW (kDa)^a^ | Experimental MW (kDa)^b^ | Number of unique peptides matched | Protein sequence coverage (%) | Score^c^ | Observed (m/z) | Partial sequence obtained^d^ |
| --- | --- | --- | --- | --- | --- | --- | --- | --- | --- |
| 1st | Uncharacterized protein LOC107439767 of *Parasteatoda tepidariorum* | XP_015907950.1 | 212.279 | >97 | 6 | 4.5 | 263 | 471.924  707.395  742.929  742.931  772.418  713.418 | QQALETVQNNIR  QQALETVQNNIR  TLLATQFEATYAR  TLLATQFEATYAR  EISEIFDAISYEK  VIAHELVHQWFGNLVTMK |
| 1st | Apolipophorins of *Parasteatoda tepidariorum* | XP_021003806.1 | 366.911 | >97 | 2 | 0.6 | 94 | 588.86  613.896 | NYVELSLPNK  TVEVFLSYIR |
| 2nd | α-latroinsectotoxin precursor of *Latrodectus tredecimguttatus* | CAA78464.1 | 158.449 | >97 | 8 | 5.2 | 287 | 411.717  448.761  489.245  591.81  599.822  641.86  682.888  686.874 | GSQVEFR  IVFQDFK  EIVFDINK  IVQYFNNER  YAIQFEQDGK  TFFDLAIENGR  NEEIPFFLVEK  NEYPFYLAVEK |
| 2nd | δ-latroinsectotoxin precursor of *Latrodectus tredecimguttatus* | CAA63363.1 | 136.137 | >97 | 4 | 3.9 | 134 | 516.339  531.303  502.954  508.249 | LVLETIESK  NTAALEEVSK  TGEGYTSLHIAAMR  TGEGYTSLHIAAMR |
| 2nd | α-latrocrustotoxin precursor  Proteins matching the same set of peptides;  α-latrocrustotoxin precursor of *Latrodectus tredecimguttatus* | Q9XZC0.2  AAD33043.1 | 158.752  156.678 | >97 | 3 | 2.9 | 111 | 545.866  478.622  688.736 | VSILNYLIR  TSEDGSLHSLLFK  IINQELAIPNNAADNNAIR |
| 2nd | α-latrotoxin of *Latrodectus hesperus*  Proteins matching the same set of peptides;  α-latrotoxin of *Latrodectus geometricus*  α-latrotoxin of *Latrodectus hesperus*  α-latrotoxin of *Latrodectus pallidus* | AGD80166.1  AGD80170.1  AGD80171.1  AGD80172.1 | 154.923  153.966  155.072  153.013 | >97 | 4 | 3.8 | 97 | 587.324  636.395  470.625  735.754 | DITTPIGDWR  NDWPVASTLLR  LEEPNGILLHFK  LDIEQTLLGCSDLPFDQIK |
| 2nd | α-latrotoxin precursor of *Latrodectus tredecimguttatus* | CAA38753.1 | 157.386 | >97 | 4 | 3.6 | 73 | 515.839  587.324  470.625  735.754 | VLQVLMTVK  DITTPIGDWR  LEEPNGILLHFK  LDIEQTLLGCSDLPFDQIK |
| 3rd | Hemocyanin C chain of *Latrodectus hesperus* | ADV40153.1 | 37.976 | 69.03 | 12 | 52.0 | 410 | 572.342  763.449  509.304  537.969  806.454  853.473  853.476  569.323  569.336  589.992  607.643  660.644 | FFPVETLYK  GVTLPPVQEVFPDK  GVTLPPVQEVFPDK  VLPLFEYCNLDTK  VLPLFEYCNLDTK  ILDAINLGYVTDADGR  ILDAINLGYVTDADGR  ILDAINLGYVTDADGR  ILDAINLGYVTDADGR  RVLPLFEYCNLDTK  LFSSFQQDHLEEANR  KGEMFYYMHQQMCAR |
| 3rd | Hemocyanin C chain of *Parasteatoda tepidariorum* | XP_015914467.1 | 78.587 | 69.03 | 13 | 18.7 | 377 | 445.256  453.241  467.269  478.722  494.773  494.786  494.821  572.342  661.335  669.33  763.449  509.304  607.643 | LMIELDR  LMIELDR  KFEQLLR  SMGFPFDR  DLVGQLTDK  DLVGQLTDK  DLVGQLTDK  FFPVETIYK  FIDDMFNEYK  FIDDMFNEYK  GISLPPVQEVFPDK  GISLPPVQEVFPDK  LFSSFQQDHLIEANR |
| 3rd | Hemocyanin C chain of *Stegodyphus mimosarum* | KFM75964.1 | 72.640 | 69.03 | 7 | 9.4 | 204 | 445.256  453.241  470.74  478.722  572.342  661.335  669.33 | LMIELDR  LMIELDR  AMGFPFDR  AMGFPFDR  FFPVETIYK  FIDDMFNEYK  FIDDMFNEYK |
| 3rd | Hemocyanin F chain of *Stegodyphus mimosarum* | KFM66956.1 | 72.541 | 69.03 | 7 | 11.1 | 158 | 456.233  470.74  478.722  485.256  658.86  784.469  523.337 | FFIELDK  AMGFPFDR  AMGFPFDR  FHAELAPGK  FIDNIFQEYK  GVTVPPIQEIFPDR  GVTVPPIQEIFPDR |
| 3rd | Hemocyanin subunit F of *Nephila inaurata madagascariensis* | CAD68056.1 | 72.326 | 69.03 | 6 | 9.9 | 152 | 456.233  478.722  485.256  658.86  784.469  523.337 | FFIELDK  SMGFPFDR  FHAELAPGK  FIDNIFQEYK  GVTVPPIQEIFPDR  GVTVPPIQEIFPDR |
| 3rd | Hemocyanin subunit D of *Nephila inaurata madagascariensis* | CAD68054.1 | 72.483 | 69.03 | 4 | 6.4 | 117 | 433.719  436.757  736.948  491.699 | YDELGNR  LVLEDQR  DAYLELSHGINLK  DAYLELSHGINLK |
| 3rd | α-latroinsectotoxin precursor of *Latrodectus tredecimguttatus* | CAA78464.1 | 158.449 | >97 | 4 | 2.9 | 113 | 411.735  591.808  682.89  686.99 | GSQVEFR  IVQYFNNER  NEEIPFFLVEK  NEYPFYLAVEK |
| 3rd | Hemocyanin subunit F of *Euphrynichus bacillifer* | CCA94920.1 | 72.485 | 69.03 | 4 | 4.8 | 108 | 433.719  456.233  470.74  478.722 | YDELGNR  FFIELDK  AMGFPFDR  AMGFPFDR |
| 3rd | Hemocyanin subunit D of *Latrodectus hesperus* | ADV40138.1 | 38.235 | 69.03 | 3 | 10.5 | 103 | 462.338  526.252  784.489 | EVPLDLER  FVPAETINR  GLDILGALIESSYESLNK |
| 3rd | Hemocyanin G chain of *Stegodyphus mimosarum* | KFM73503.1 | 69.722 | 69.03 | 3 | 4.9 | 88 | 433.719  462.338  784.489 | YDELGNR  LLGVGVLPR  GVTLPPIQEVFPDR |
| 3rd | Hemocyanin AA6 chain | P80476.1 | 72.197 | 69.03 | 4 | 6.2 | 67 | 470.74  478.722  523.806  784.489 | AMGFPFDR  AMGFPFDR  FVPAETINR  GITVPPIQEVFPDR |
| 3rd | Hemocyanin B chain of *Stegodyphus mimosarum* | KFM59357.1 | 74.335 | 69.03 | 3 | 4.0 | 57 | 470.74  478.722  645.4 | AMGFPFDR  AMGFPFDR  FIDDLFQTYK |
| 4th | Hemocyanin F chain of *Parasteatoda tepidariorum* | XP_015923305.1 | 66.818 | 67.607 | 3 | 5.3 | 226 | 460.749  658.864  770.475 | ATLPIYDK  FIDNIFQEYK  ILPLFEHLTSLTR |
| 5th | Chitinase of *Araneus ventricosus* | AAN39100.1 | 47.608 | 53.251 | 1 | 2.3 | 60 | 589.327 | ENWGLGAFQR |
| 6th | Astacin-like metalloprotease toxin of *Stegodyphus mimosarum* | KFM58572.1 | 46.075 | 48.759 | 11 | 40 | 361 | 696.874  464.921  704.866  718.404  479.275  823.384  549.265  591.957  665.702  665.709  694.738 | SIMLYGEYAFAK  SIMLYGEYAFAK  SIMLYGEYAFAK  LTIVTGCWSSVGR  LTIVTGCWSSVGR  GGEQEISLSEGCHDK  GGEQEISLSEGCHDK  KGGEQEISLSEGCHDK  LKPWENNLLGEEFDYK  LKPWENNLLGEEFDYK  EGVVIGLINNKPGLSDSDVR |
| 6th | Astacin-like metalloprotease toxin 1 of *Parasteatoda tepidariorum* | XP_015917054.1 | 46.392 | 48.759 | 9 | 29.5 | 281 | 696.874  464.921  704.866  793.893  529.609  529.613  823.384  549.265  591.957 | SIMLYGEYAFAK  SIMLYGEYAFAK  SIMLYGEYAFAK  DILQEAFDEYESK  DILQEAFDEYESK  DILQEAFDEYESK  GGEQEISLSEGCHDK  GGEQEISLSEGCHDK  KGGEQEISLSEGCHDK |
| 6th | Astacin-like metalloprotease toxin 1 of *Parasteatoda tepidariorum* | XP_021000929.1 | 45.765 | 48.759 | 7 | 22.6 | 232 | 506.736  696.874  464.921  704.866  823.384  549.265  591.957 | LYECNGEK SIMLYGEYAFAK  SIMLYGEYAFAK  SIMLYGEYAFAK  GGEQELSLSEGCHDK  GGEQELSLSEGCHDK  KGGEQELSLSEGCHDK |
| 6th | Arginine kinase of *Parasteatoda tepidariorum* | XP_015928377.1 | 46.76 | 49.536 | 5 | 14.2 | 138 | 429.751  468.245  578.322  574.604  609.702 | VLEDIAAK  ASVHIALPK  MGLTEYQAVK  GEHTESEGGVYDISNK  LGYLTFCPTNLGTTIR |
| 7th | Hemocyanin subunit D of *Latrodectus hesperus* | ADV40138.1 | 38.235 | 39.972 | 5 | 17.4 | 225 | 523.786  560.764  599.345  818.978  893.528 | FVPAETINR  EVDVQDMER  DFDDFINLAK  NLTALSPEPLPEAER  TGNILDPEYNLAYFR |
| 7th | Hemocyanin subunit C of *Latrodectus hesperus* | ADV40153.1 | 37.976 | 39.972 | 2 | 8.2 | 70 | 763.453  806.418 | GVTLPPVQEVFPDK  VLPLFEYCNLDTK |
| 7th | Putative serine protease of *Latrodectus hesperus* | ADV40282.1 | 38.728 | 39.972 | 3 | 7.4 | 57 | 469.233  469.249  618.33 | TQYEQIR  TQYEQIR  VISYPDLEGDK |

^a^ Theoretical molecular weight (MW) obtained after LC-MS/MS analysis

^b^ Experimental MW was from calculation

^c^ Score XC obtained after LC-MS/MS

^d^ Observed (m/z) obtained from LC-MS/MS

^e^ Partial sequence obtained from MASCOT search
