# Supplementary material for: Partial proteomic analysis of brown widow spider (Latrodectus geometricus) venom to determine the biological activities
Source: Toxicon X. 2020 Oct 24;8:100062. doi: 10.1016/j.toxcx.2020.100062 (PMC7607507; doi:10.1016/j.toxcx.2020.100062)
Supplement: Multimedia component 2 [file mmc2.docx]

Supplementary Table 2. Protein identification of *Latrodectus geometricus* venom using RP-HPLC fraction

| Fraction no. | Protein name | Accession no. | Theoretical MW (kDa)^a^ | Experimental MW (kDa)^b^ | Number of unique peptides matched | Protein sequence coverage (%) | Score^c^ | Observed (m/z)^d^ | Partial sequence obtained^e^ |
| --- | --- | --- | --- | --- | --- | --- | --- | --- | --- |
| 5 | Astacin-like metalloprotease toxin 1 of *Parasteatoda tepidariorum* | XP_015917054.1 | 46.392 | 32.539 | 9 | 29.5 | 281 | 696.874  464.921  704.866  793.893  529.609  529.613  823.384  549.265  591.957 | SIMLYGEYAFAK  SIMLYGEYAFAK  SIMLYGEYAFAK  DILQEAFDEYESK  DILQEAFDEYESK  DILQEAFDEYESK  GGEQEISLSEGCHDK  GGEQEISLSEGCHDK  KGGEQEISLSEGCHDK |
| 6 | Arginine kinase of *Parasteatoda tepidariorum* | XP_015928377.1 | 46.76 | 49.536 | 5 | 14.2 | 138 | 429.751  468.245  578.322  574.604  609.702 | VLEDIAAK  ASVHIALPK  MGLTEYQAVK  GEHTESEGGVYDISNK  LGYLTFCPTNLGTTIR |
| 8 | Putative serine protease of *Latrodectus hesperus* | ADV40282.1 | 38.728 | 20.549 | 3 | 7.4 | 57 | 469.233  469.249  618.33 | TQYEQIR  TQYEQIR  VISYPDLEGDK |
| 8 | Chitinase of *Araneus ventricosus* | AAN39100.1 | 47.608 | 18.592 | 1 | 2.3 | 60 | 589.327 | ENWGLGAFQR |
| 9 | Astacin-like metalloprotease toxin of *Stegodyphus mimosarum* | KFM58572.1 | 46.075 | 32.539 | 11 | 40 | 361 | 696.874  464.921  704.866  718.404  479.275  823.384  549.265  591.957  665.702  665.709  694.738 | SIMLYGEYAFAK  SIMLYGEYAFAK  SIMLYGEYAFAK  LTIVTGCWSSVGR  LTIVTGCWSSVGR  GGEQEISLSEGCHDK  GGEQEISLSEGCHDK  KGGEQEISLSEGCHDK  LKPWENNLLGEEFDYK  LKPWENNLLGEEFDYK  EGVVIGLINNKPGLSDSDVR |
| 11 | α-Latroinsectotoxin precursor of *Latrodectus tredecimguttatus* | CAA78464.1 | 158.449 | >97 | 8 | 5.2 | 287 | 411.717  448.761  489.245  591.81  599.822  641.86  682.888  686.874 | GSQVEFR  IVFQDFK  EIVFDINK  IVQYFNNER  YAIQFEQDGK  TFFDLAIENGR  NEEIPFFLVEK  NEYPFYLAVEK |
| 11 | U24-ctenitoxin-Pn1a of *Trichonephila clavipes* | PRD29014.1 | 116.247 | >97 | 6 | 40.6 | 100 | 496.221  496.229  496.243  496.259  774.386  516.595 | FCVCYDK  FCVCYDK  FCVCYDK  FCVCYDK  ENGDYEELQCYK  ENGDYEELQCYK |
| 12 | Venom allergen antigen 5-like protein of *Dirofilaria immitis* | AAB62535.1 | 56.6291 | 35.295 | 1 | 4.1 | 54 | 529.369 | KNIVTQINK |
| 12 | δ-Latroinsectotoxin precursor of *Latrodectus tredecimguttatus* | CAA63363.1 | 136.137 | >97 | 4 | 3.9 | 134 | 516.339  531.303  502.954  508.249 | LVLETIESK  NTAALEEVSK  TGEGYTSLHIAAMR  TGEGYTSLHIAAMR |
| 13 | α -Latrocrustotoxin precursor of *Latrodectus tredecimguttatus* | Q9XZC0.2 | 158.752 | >97 | 3 | 2.9 | 111 | 545.866  478.622  688.736 | VSILNYLIR  TSEDGSLHSLLFK  IINQELAIPNNAADNNAIR |
| 13 | α -Latrotoxin of *Latrodectus hesperus* | AGD80166.1 | 154.923 | >97 | 4 | 3.8 | 97 | 587.324  636.395  470.625  735.754 | DITTPIGDWR  NDWPVASTLLR  LEEPNGILLHFK  LDIEQTLLGCSDLPFDQIK |
| 14 | α -Latrotoxin precursor of *Latrodectus tredecimguttatus* | CAA38753.1 | 157.386 | >97 | 4 | 3.6 | 73 | 515.839  587.324  470.625  735.754 | VLQVLMTVK  DITTPIGDWR  LEEPNGILLHFK  LDIEQTLLGCSDLPFDQIK |

^a^ Theoretical molecular weight (MW) obtained after LC-MS/MS analysis

^b^ Experimental MW was from calculation

^c^ Score XC obtained after LC-MS/MS

^d^ Observed (m/z) obtained from LC-MS/MS

^e^ Partial sequence obtained from MASCOT search
